# A 7 Year Summary of Emergency Department Visits by Patients With Mental Health Disorders

**DOI:** 10.3389/fpsyt.2022.831843

**Published:** 2022-02-09

**Authors:** Danielle Brathwaite, Anna E. Waller, Bradley N. Gaynes, Rachel Stemerman, Tracy M. Deselm, Jason J. Bischof, Judith Tintinalli, Jane H. Brice, Montika Bush

**Affiliations:** ^1^Department of Health Policy and Management, University of North Carolina Gillings School of Global Public Health, Chapel Hill, NC, United States; ^2^Department of Emergency Medicine, University of North Carolina School of Medicine, Chapel Hill, NC, United States; ^3^Carolina Center for Health Informatics, University of North Carolina, Chapel Hill, NC, United States; ^4^Department of Psychiatry, University of North Carolina School of Medicine, Chapel Hill, NC, United States; ^5^Department of Epidemiology, University of North Carolina Gillings School of Global Public Health, Chapel Hill, NC, United States; ^6^Department of Emergency Medicine, The Ohio State University Wexner Medical Center, Columbus, OH, United States

**Keywords:** emergency department, emergency services, behavioral health, mental health, syndromic surveillance

## Abstract

**Objectives:**

Emergency departments (EDs) have been increasingly utilized over time for psychiatric care. While multiple studies have assessed these trends in nationally representative data, few have evaluated these trends in state-level data. This investigation seeks to understand the mental health-related ED burden in North Carolina (NC) by describing trends in ED visits associated with a mental health diagnosis (MHD) over time.

**Methods:**

Using data from NC DETECT, this investigation describes trends in NC ED visits from January 1, 2008 through December 31, 2014 by presence of a MHD code. A visit was classified by the first listed MHD ICD-9-CM code in the surveillance record and MHD codes were grouped into related categories for analysis. Visits were summarized by MHD status and by MHD category.

**Results:**

Over 32 million ED visits were recorded from 2008 to 2014, of which 3,030,746 (9.4%) were MHD-related visits. The average age at presentation for MHD-related visits was 50 years (SD 23.5) and 63.9% of visits were from female patients. The proportion of ED visits with a MHD increased from 8.3 to 10.2% from 2008 to 2014. Annually and overall, the largest diagnostic category was stress/anxiety/depression. Hospital admissions resulting from MHD-related visits declined from 32.2 to 18.5% from 2008 to 2014 but remained consistently higher than the rate of admissions among non-MHD visits.

**Conclusion:**

Similar to national trends, the proportion of ED visits associated with a MHD in NC has increased over time. This indicates a need for continued surveillance, both stateside and nationally, in order to inform future efforts to mitigate the growing ED burden.

## Introduction

Emergency departments (EDs) in the US have been increasingly utilized for psychiatric care (1–7). This increase in the number of mental health-related ED visits has been demonstrated among adult (1–3) and pediatric (4, 6, 7) populations, as well as across individual diagnoses such as depression (8), anxiety (9), substance abuse (10, 11), and suicidal ideation (12–15). The consistency of results across different national datasets, including the National Hospital Ambulatory Medical Care Survey (NHAMCS) (2–7, 9, 12) and the Nationwide Emergency Department Sample (NEDS) (1, 8) suggests a growing crisis, but remaining gaps in the literature must be addressed before surveillance trends can be translated to targeted improvements in patient care and health policy. The majority of published studies have relied upon national data, and more recent studies have summarized mental health-related ED utilization through 2015 (4) or even 2018, (15) but few have conducted comprehensive evaluations of data from individual states. One of the only states that has published data on this issue is North Carolina (NC). The North Carolina Disease Event Tracking and Epidemiologic Collection Tool (NC DETECT), a state-wide public health surveillance system, has previously been used to assess psychiatric ED utilization prior to 2010 (16, 17). In order to (1) continue monitoring the situation at the state level, and (2) keep up with nationally published data, there is a need to update the NC-specific information available in the literature.

In an update of previously published data, this investigation characterizes those patients with the presence of a mental health diagnosis (MHD) code during an ED visit in NC and assesses patterns among ED visits in grouped diagnostic categories, in an effort to further explain previously established trends. Results will provide insight into how best to tailor ED-based mental health resources in the future, particularly within the state of NC.

## Materials and Methods

### Study Design, Data Source, and Population

This is a retrospective study of healthcare encounters obtained from a data use agreement for statewide ED surveillance information. NC DETECT includes near real-time information about ED visits throughout the state of NC from all civilian, acute care, hospital-affiliated EDs (~124 sites) (18). ED data from NC DETECT includes visit-level information from all patients treated in an ED during the study period, but excludes those who are directly admitted or triaged to care at other clinical sites (18). The available data contain information on patient demographics as well as specific information from the medical record, including the chief complaint, triage notes, disposition status, disposition diagnosis descriptions, and International Classification of Diseases, Clinical Modification (ICD-CM) diagnosis codes entered upon discharge (18). All ED visits from January 2008 through December 2014 (*N* = 32,121,296) were included in this investigation. This time frame was selected in order to accommodate the transition to ICD-10-CM in October 2015. Data from 2015 was found to be inconsistent within MHD-related categories likely due to the transition and was thus dropped from this investigation. The UNC Office of Human Research Ethics determined this study did not constitute humans subject research; therefore, did not require Institutional Review Board approval.

### Identification of MHD-Related Visits

A MHD flag was generated using the first MHD ICD-9-CM code reported under the listed discharge diagnoses for each visit. The flag was not set if no MHD codes were present for any listed discharge diagnoses. MHD-related visits were grouped into the following categories: 1) Stress/Anxiety/Depression, 2) Schizophrenia/Delusional/Psychosis/Dementia, 3) Bipolar Disorder, 4) Suicidal/Homicidal Ideation, 5) Personality/Conduct Disorder, 6) Psychiatric Examination, 7) Mental Disorders from Brain Damage, 8) Development Disorders Originating in Childhood, 9) Eating Disorders, and 10) Miscellaneous/other. Diagnostic categories were updated from the previously published work with NC DETECT data based upon further clinical review (17). Collaborating physicians made small alterations to the ICD-9-CM codes used to define Bipolar Disorder (codes 296.2 and 296.3 were reclassified under Depression), as well as combined the categories for Dementia and Schizophrenia/Delusional/Psychosis to reduce potential misclassifications between disorder types after the introduction of a new ICD-9-CM code for Delirium in 2012. Otherwise, the coding scheme matched the one published by Hakenewerth et al. (17). Consistent with Hakenewerth et al. (17) substance use was not included under the list of MHD categories.

### Data Analysis

Descriptive statistics were used to summarize patient demographics, expected sources of payment, transportation modes, and discharge dispositions of both non-MHD and MHD-related ED visits. Standardized differences were computed to detect an imbalance by non-randomized MHD status (19, 20). A 10% standardized difference between groups was considered meaningful (19). Visit counts were summarized by MHD status and by diagnostic category, including visits with multiple MHD ICD-9-CM diagnosis codes listed in the record. Visits resulting in a hospital admission were summarized by MHD status and by diagnostic category. Plots were generated to visually represent trends in MHD-related ED visits over time. This investigation focused on three specific time trends: (1) the proportion of ED visits associated with a MHD, (2) the proportion of MHD-related visits attributed to each of the top four diagnostic categories, and (3) the proportion of ED visits resulting in a hospital admission by MHD status. All analyses were completed using SAS 9.4 (Cary, NC).

## Results

**Figure 1:**
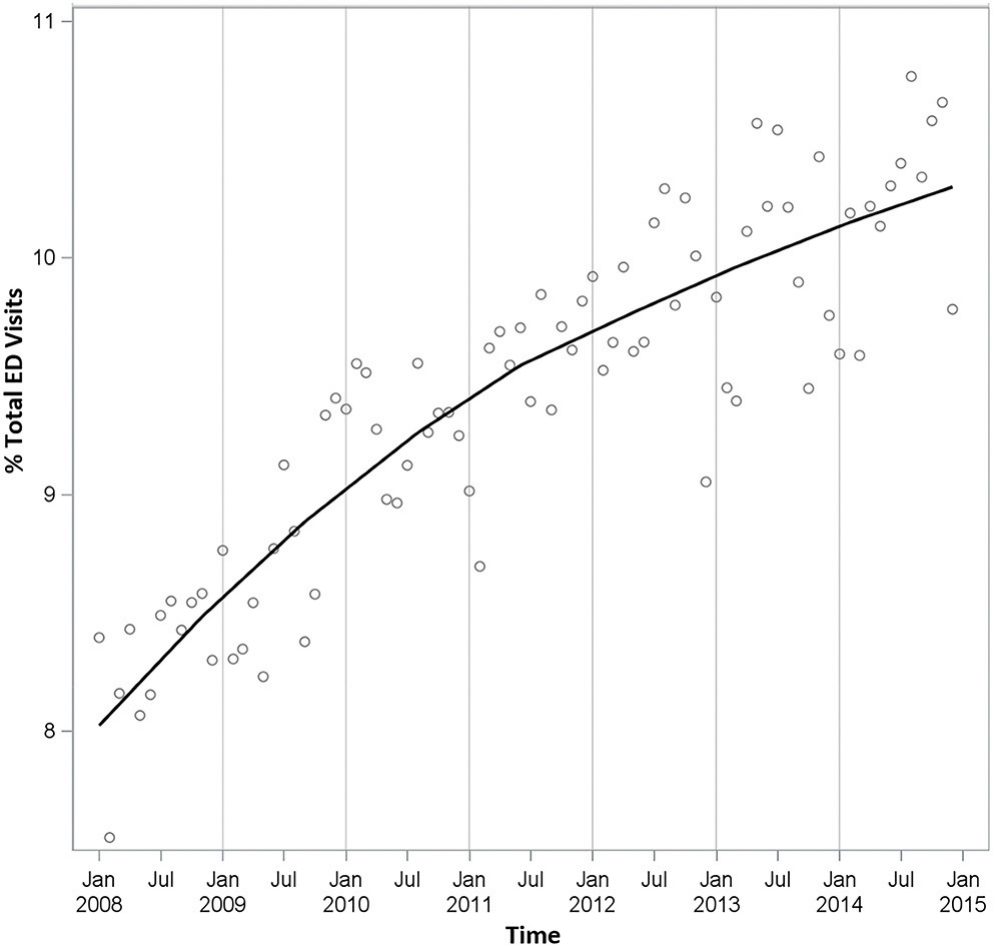
Proportion of total ED visits associated with a mental health diagnosis (MHD).

We observed 32,121,296 ED visits across the state of NC between 2008 and 2014 with a MHD flag associated with 3,030,746 (9.4%) of these visits (Table 1). For ED visits without a MHD-related code, the average age at presentation was 38 years (SD 26.2), 55.8% of visits were from female patients, 17.4% of visits were insured through Medicare, and 12.1% of visits had patients arrive via ambulance. In contrast, for MHD-related ED visits, patients were older (50 years, SD 23.5) and visits were more likely to be by female patients (63.9%), more heavily insured by Medicare (34.5%), and more likely to have patients arrive by ambulance (26.8%). A larger percentage of MHD-related ED visits were associated with self-harm and substance abuse ICD-9-CM codes when compared to non-MHD visits. Both MHD and non-MHD related ED visits increased between 2008 and 2014, but not symmetrically (Table 1). MHD-related visits saw a steady annual increase resulting in a total change of 43.8% between 2008 and 2014 while non-MHD visits only increased 14.5% during the study period. The relative proportion of MHD-related ED visits to the total number of ED visits in a given year increased from an annual average of 8.3% in 2008 to 10.2% in 2014 (Figure 1).

**Table 1:** Emergency department visit characteristics by presence of a MHD-related code.

**Characteristic**	**Non-MHD visits**		**MHD visits**		**Overall**		**Standardized difference**
**Total visits (*N*)**	29,090,550		3,030,746		32,121,296		
**Mean age (SD)**	37.7 ± 26.22		49.8 ± 23.53		38.8 ± 26.22		0.49^*^
	***N***	**%**	***N***	**%**	***N***	**%**	
**Age group**							
0–11	4,326,903	14.9	35,417	1.2	4,362,320	13.6	−0.52^*^
12–17	1,541,532	5.3	106,961	3.5	1,648,493	5.1	−0.09
18–24	3,753,077	12.9	263,336	8.7	4,016,413	12.5	−0.14^*^
25–44	8,685,861	29.9	946,449	31.2	9,632,310	30.0	0.03
45–64	6,395,692	22.0	878,191	29.0	7,273,883	22.6	0.16^*^
≥65	4,387,485	15.1	800,392	26.4	5,187,877	16.2	0.28^*^
**Female**	16,224,986	55.8	1,936,472	63.9	18,161,458	56.5	0.17^*^
**Insurance type**							
Medicare	5,048,781	17.4	1,045,380	34.5	6,094,161	19.0	0.40^*^
Medicaid	6,787,098	23.3	593,643	19.6	7,380,741	23.0	−0.09
Private	6,809,368	23.4	546,412	18.0	7,355,780	22.9	−0.13^*^
Self-Pay	6,829,701	23.5	527,127	17.4	7,356,828	22.9	−0.15^*^
Other	2,624,572	9.0	240,510	7.9	2,865,082	8.9	−0.04
**Transport mode**							
Ambulance	3,516,999	12.1	813,301	26.8	4,330,300	13.5	0.38^*^
Walk-in	18,966,520	65.2	1,599,906	52.8	20,566,426	64.0	−0.25^*^
Other	1,319,000	4.5	108,202	3.6	1,427,202	4.4	−0.05
**Injury**							
Any Injury	6,117,828	21.0	562,626	18.6	6,680,454	20.8	−0.06
Self-Harm	19,359	0.1	61,803	2.0	81,162	0.3	0.19^*^
Assault	248,133	0.9	22,962	0.8	271,095	0.8	−0.01
**ED disposition**							
Discharged home	23,113,037	79.5	1,827,528	60.3	24,940,565	77.6	−0.43^*^
Admitted	2,974,231	10.2	808,761	26.7	3,782,992	11.8	0.44^*^
Transferred	495,369	1.7	180,177	5.9	675,546	2.1	0.22^*^
Eloped	935,273	3.2	44,242	1.5	979,515	3.0	−0.11^*^
Other	456,092	1.6	64,711	2.1	520,803	1.6	0.037
**Substance abuse**	634,391	2.2	350,192	11.6	984,583	3.1	0.38^*^
**Annual visit count**							
2008	3,843,091		347,790		4,190,881		N/A
2009	4,000,311		381,700		4,382,011		N/A
2010	3,996,392		409,276		4,405,668		N/A
2011	4,157,944		436,365		4,594,309		N/A
2012	4,353,369		473,823		4,827,192		N/A
2013	4,340,661		481,686		4,822,347		N/A
2014	4,398,782		500,106		4,898,888		N/A

MHD, Mental Health Diagnosis-Related; ^*^Indicates a standardized difference >|0.10|. Categories may not total 100% given missing data or “unknown” fields.

**Figure 2:**
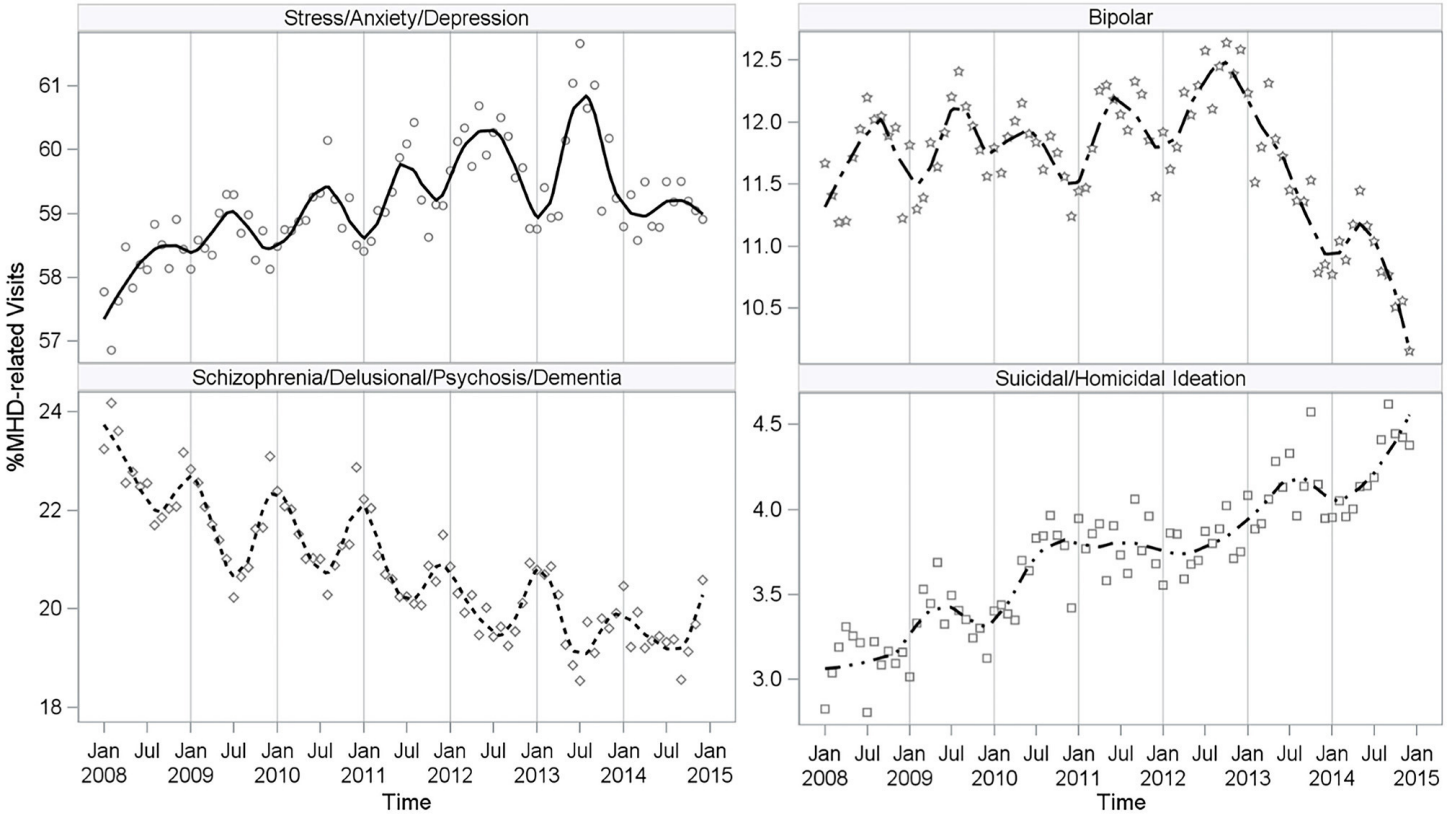
Proportion of MHD Visits by Most Prevalent Diagnostic Categories. MHD-Related, Mental Health Diagnosis-Related.

The four most common diagnostic categories of MHD-related visits were 1) Stress/Anxiety/Depression, 2) Schizophrenia/Delusional/Psychosis/Dementia, 3) Bipolar Disorder, and 4) Suicidal/Homicidal Ideation, together representing 95% of MHD-related ED visits during the study period (Supplementary Table 1). Less than 10% of Stress/Anxiety/Depression visits had multiple MHD codes per visit while almost 60% of Suicidal/Homicidal Ideation visits had multiple MHD codes per visit. The proportion of MHD-related ED visits with more than one MHD code rose from 15.0 to 17.9% from 2008 to 2014 (data not shown). Time trends in all four categories demonstrated some level of seasonality with visits peaking cyclically during certain months of the year (Figure 2). Among the four most common MHD categories, an overall increase in the proportion of MHD-related ED visits was observed with mood disorders or acute psychiatric crises in which patients may pose a harm to themselves or others (Figure 2). The proportion of MHD-related visits associated with Stress/Anxiety/ Depression increased slightly over time from an annual average of 58.1% in 2008 to 59.1% in 2014, with a peak at over 60% of visits in 2013. The proportion of MHD-related visits associated with Suicidal/Homicidal Ideation similarly increased from an annual average of 3.1% in 2008 to 4.2% in 2014. Of note, the trend line associated with Suicidal/Homicidal Ideation demonstrated a weaker cyclical pattern, supporting a consistent increase in the proportion of visits over time (Figure 2). Moving in the opposite direction, the proportion of visits associated with Schizophrenia/ Delusional/Psychosis/Dementia decreased over time from an annual average of 22.7% in 2008 to 19.5% in 2014, and the proportion of visits associated with Bipolar Disorder peaked at an annual average of 12.2% in 2012 and then dropped steeply to 10.9% in 2014.

**Figure 3:**
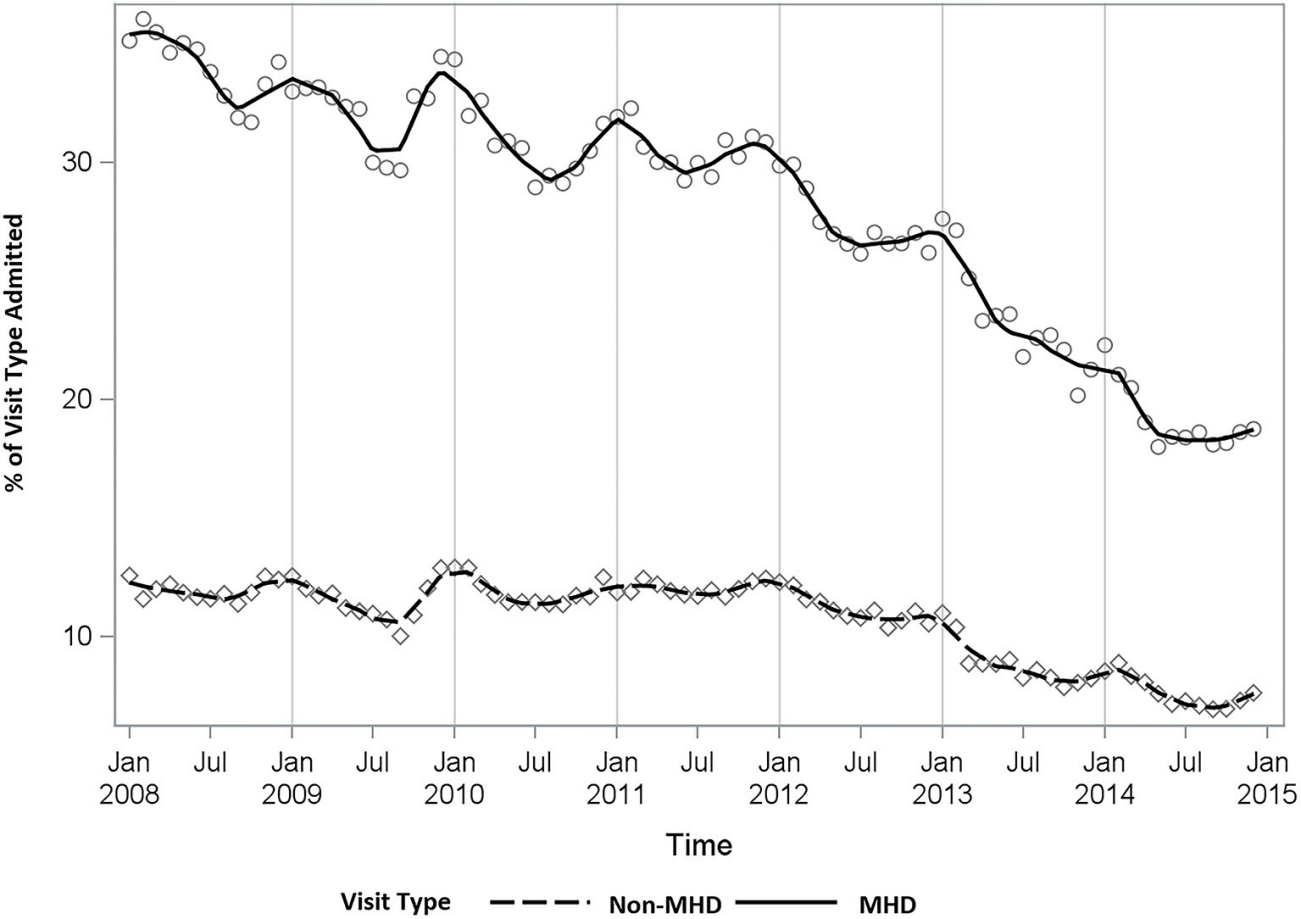
Proportion of ED visits resulting in a hospital admission (MHD vs. Non-MHD). MHD, Mental Health Diagnosis.

Although ED visits increased over the study period, the admission rate declined from an annual average of 32.2% in 2008 to 18.5% in 2014 among MHD-related visits and from 11.3 to 7.3% in non-MHD related visits (Figure 3). Seasonal peaks are more noticeable among the MHD-related visits with higher rates occurring in January and lower rates in July. Despite Stress/Anxiety/Depression representing over 50% of MHD-related visits, a higher proportion of Schizophrenia/Delusional/Psychosis/Dementia patients were admitted to the hospital (Supplementary Table 1). Visits related to Schizophrenia/Delusional/ Psychosis/Dementia or Suicidal/Homicidal Ideation had the highest average admission proportion (≥30%) (Supplementary Table 1). On average, the lowest admission rates ( ≤ 13%) were observed among visits with an MHD category of Mental Disorders from Brain Damage or Development Disorders Originating in Childhood (Supplementary Table 1). Overall MHD-related ED visits and the resulting hospital admissions demonstrated different diagnostic trends. Understanding those trends among patients who present to the ED will be important to improving ED-based care and health policy.

## Discussion

Emergency departments across NC have seen an increase in visits associated with a mental health diagnosis over time. The proportion of MHD-related ED visits increased by 23% from 2008 to 2014, and trends suggest this upward trajectory will continue. Total ED utilization has increased over time, and so it is reasonable to expect that mental health-specific visits would increase in parallel. Instead, the rate of increase among MHD-related visits is three times that of non-MHD visits. Additionally, an increase in visits associated with multiple mental health diagnoses was identified. This investigation also found characteristic differences between non-MHD and MHD-related ED visits. When compared to non-MHD visits, MHD-related visits were more likely to occur among older female patients (≥65 years) and were more likely to be paid for by Medicare. This result is important for hospital administrations considering the resources needed to initiate or advance Geriatric EDs (21). Patients with associated MHD-related visits were more likely to arrive via ambulance and be admitted to the hospital, suggesting that patients were more likely to present in crisis. Given the rate of increase in MHD-related visits when compared to non-MHD visits and the unique characteristics that differentiate MHD-related visits from non-MHD visits, EDs must focus on mental health services in addition to other competing priorities to improve care for this particular patient population.

This investigation demonstrated that NC EDs are seeing an overall decline in the rate of admissions resulting from patient visits. Admission rates saw a much larger decline among MHD-related visits when compared to non-MHD visits. This is an interesting contrast–that despite an increase in the number of visits, there has been a decrease in admissions. While not investigated here, this paradox may be attributed to limited inpatient resources, difficulties in coordinating care outside of the ED, or an increase in visits associated with patients who may not require inpatient care, such as those with mood disorders but not in active crisis. Of note, the admissions observed here do not differentiate between psychiatric and medical admissions as there is no qualifier in the data to signify admission unit or service.

Trends similar to our findings have been observed in previously published studies looking at the mental health ED burden at the national level (1–7). While NC EDs are experiencing those same trends, this investigation extends the conversation through an analysis of trends based on specific diagnostic categories. The majority of mental health ED visits are attributed to mood disorders (stress/anxiety/depression) throughout the study period, with an upward trend over time. As a result, as EDs look to cater mental health services to particular diagnostic groups, special focus should be placed on mood disorders. In contrast, the proportion of visits associated with psychotic disorders or dementia have been on the decline. Most concerning though is the small but steep increase in the proportion of visits associated with suicidal or homicidal ideation (SI/HI). Although this category is only 4% of MHD-related visits, over 30% of visits in this diagnosis category resulted in admission. This shift in diagnostic prevalence suggests a potential increase in the acuity of these SI/HI patients presenting to EDs, further supported by our finding that these patients are also more likely to arrive via ambulance.

If EDs are to prioritize bolstering mental health resources, then specifically targeting resources to support patients with suicidal/homicidal ideation may have the greatest benefit in reducing admissions and would cater to the most rapidly increasing diagnostic group. It is also important to note that the ED is not a source of treatment for patients with SI/HI, but rather a place to ensure safety and help connect patients with outpatient and inpatient resources designed to provide psychiatric treatment for SI/HI. Thus, if this patient group continues to grow, not just ED resources, but both inpatient and outpatient psychiatric resources across the state of NC need to be strengthened in order to truly help these patients. It is also important to consider that ED utilization has increased secondary to the closure or absence of available outpatient resources for patients with higher acuity concerns, such as suicidal ideation. Thus, moving forward, this problem needs to be addressed from both a perspective of bolstering outpatient resources and strengthening existing ED resources.

Given the observational design of this investigation, a pragmatic next step would be to consider an intervention-driven experimental design aimed at reducing MHD-related ED visits among the identified prevalent patient populations–older adults, those with mood disorders, and/or those presenting with SI/HI. Non-observational randomized trials have looked to reduce post-hospitalization ED utilization among similar patient groups, including acutely injured trauma survivors and hospitalized patients with comorbid substance use disorders (22, 23). These trials employed care coordination and case management interventions to limit adverse outcomes post-hospitalization (22, 23). Similar study designs could prove useful in developing targeted interventions to prevent MHD-related ED visits across NC.

## Strengths and Limitations

NC DETECT is a syndromic surveillance data system which relies upon secondary data collected from administrative and clinical records. Despite the availability of data from 2015 onward, 2014 was chosen as the stopping point for this investigation for several reasons. First, the 2015 dataset included the transition to ICD-10-CM codes (October 1, 2015). Changes in existing codes and the development of new codes created an inconsistent coding scheme across the ICD-CM codes. Second, previous research using NC DETECT to assess mental health-related ED utilization looked at data from 2008 to 2010 (16, 17). This updated investigation serves as a stepping stone from 2010 through 2014 in order to gain a comprehensive and consistent understanding of trends across the state of NC, prior to looking at trends from 2015 onward. Reliance upon data from a single state (NC) allows a near complete sample (~99% of all NC ED visits captured) (24), whereas national studies looking at similar trends have relied upon limited yet representative samples. Thus, the results presented here are a complete context-specific picture of the psychiatric ED burden within NC and can be meaningfully applied to future intervention and policy development within the state.

This investigation provides an example for other states with an ED syndromic surveillance system to replicate and a platform from which NC-specific interventions can be developed to target the patient populations most in need within our state. To extend the generalizability of our study results, surveillance data from other states should be assessed for similar trends and compared to findings from national-level data. For example, the COVID-19 pandemic has highlighted the importance of ED-based care and the relationship between mental health care and ED utilization. Recent studies examining these trends have relied on state-level data sources in order to keep up with the rapidly changing healthcare landscape then aggregated the data across multiple states to gain a national perspective (25, 26). Aggregation of state-level syndromic surveillance data across various states would strengthen the study results and would be a next step for future work.

While reliance upon a syndromic surveillance data set does provide a nearly complete cohort of ED visits, information available to researchers to control for confounding factors is limited. The results presented here are reliant upon ICD-CM code-specific mental health case definitions, which assume these codes are recorded with diagnostic accuracy by hospital coders who rely on clinical documentation by healthcare providers. Mental health diagnosis codes assigned in an ED setting may also differ from those recorded in a psychiatric outpatient setting. Mental health-related codes may enter a patient's record as comorbidities secondary to a medical condition, such as a urinary tract infection or acute physical trauma.

For this investigation, the first listed MHD ICD-9-CM code was used to categorize visits. While this decision allowed us to define a binary status of a MHD-related visit, mental health comorbidities are underreported for the 16% of the study visits with more than one MHD code per visit. For example, a patient may have presented with both depression and suicidal ideation, but this visit would have only been counted under the first listed category, not both. This investigation attempted to improve upon previous published mental health case definitions through further review of ICD-CM codes by clinician collaborators (17). Changes were made to improve the accuracy of MHD code categorization for this investigation, but case definitions can and should be improved further by clinical chart review to verify ICD-CM code accuracy and to reduce the risk of falsely identifying MHD-related visits.

While this investigation specifically highlighted hospital admissions as a discharge disposition of interest, we were unable to differentiate between psychiatric admissions and medical admissions. Without a clear and consistent indicator of psychiatric admission among all ED sites, the term “admission” was used to describe any hospital admissions, regardless of the unit or service. The ED disposition indicators employed in this investigation also lacked information on where a patient had been transferred, and whether a patient was discharged home with appropriate follow-up care. This contextual information may strengthen future studies looking at ED disposition in relation to mental health-related ED visits, and the inclusion of a psychiatric admission indicator would strengthen the ability of studies to assess a patient's full hospital course.

## Conclusions

A growing need for tighter integration of mental and physical health services in the ED is required as the proportion of ED visits associated with a comorbid MHD is trending upward over time. Patients who present to the ED with a comorbid MHD are distinctly different than those who present with only non-MHD concerns. Time trends indicate that these patients are increasingly more likely to present with a mood disorder or suicidal/homicidal ideation, and increasingly receive multiple mental health diagnoses. As this unique patient population continues to grow, there is a need for continued surveillance and improved resources to care for them in the ED.

## Data Availability Statement

Publicly available datasets were analyzed in this study. This data can be found here: https://ncdetect.org/data-requests-for-applied-public-health-research/. The statewide, population-based data used in this study are available for applied public health research based in North Carolina. A formal data request is required.

## Author Contributions

All authors contributed to the study concept and design. MB led data acquisition and provided statistical expertise. MB and DB led data analysis and interpretation. DB led drafting of the manuscript and all authors assisted in critical revision of the manuscript draft. All authors agree to be accountable for the content of this work.

## Author Disclaimer

The North Carolina Disease Event Tracking and Epidemiologic Collection Tool (NC DETECT) is an advanced, statewide public health surveillance system. NC DETECT is supported by the North Carolina Division of Public Health through a federal Public Health Emergency Preparedness Grant and is managed through a collaboration between NC DPH and the University of North Carolina at Chapel Hill Department of Emergency Medicine's Carolina Center for Health Informatics. The findings and conclusions in this publication are those of the author(s) and do not necessarily represent the views of the North Carolina Department of Health and Human Services, Division of Public Health.

## Conflict of Interest

The authors declare that the research was conducted in the absence of any commercial or financial relationships that could be construed as a potential conflict of interest.

## Publisher's Note

All claims expressed in this article are solely those of the authors and do not necessarily represent those of their affiliated organizations, or those of the publisher, the editors and the reviewers. Any product that may be evaluated in this article, or claim that may be made by its manufacturer, is not guaranteed or endorsed by the publisher.
