# Supplementary material for: A 7 Year Summary of Emergency Department Visits by Patients With Mental Health Disorders
Source: Front Psychiatry. 2022 Feb 9;13:831843. doi: 10.3389/fpsyt.2022.831843 (PMC8863870; doi:10.3389/fpsyt.2022.831843)
Supplement: Supplementary file 1 [file Table_1.DOCX]

Supplementary Material

**Supplementary Table S1.** Summary of MHD-Related ED Visits and Hospital Admissions Time Trends by Diagnostic Category

|  | **Stress/ Anxiety/ Depression** | | **Schizophrenia/ Delusional/ Psychosis/ Dementia** | | **Bipolar** | | **Suicidal/ Homicidal Ideation** | | **Personality/ Conduct Disorder** | | **Mental Disorders from Brain Damage** | | **Development Disorders Originating in Childhood** | | **Eating**  **Disorders** | |
| --- | --- | --- | --- | --- | --- | --- | --- | --- | --- | --- | --- | --- | --- | --- | --- | --- |
| **Year** | **N** | **%** | **N** | **%** | **N** | **%** | **N** | **%** | **N** | **%** | **N** | **%** | **N** | **%** | **N** | **%** |
| **MHD ED Visits (% of total MHD visits)** | | | | | | | | | | | | | | | | |
| **Overall** | 1,794,607 | 59.2 | 627,529 | 20.7 | 354,110 | 11.7 | 114,096 | 3.8 | 34,991 | 1.2 | 18,229 | 0.6 | 21,495 | 0.7 | 4,222 | 0.1 |
| **>1 MHD Code per ED Visit (% of total category visits)** | | | | | | | | | | | | | | | | |
| **Overall** | 177,640 | 9.9 | 103,990 | 16.6 | 101,147 | 28.6 | 66,577 | 58.4 | 17,223 | 49.2 | 1,850 | 10.1 | 2,387 | 11.1 | 1,092 | 25.9 |
| **MHD ED Visits Resulting in Hospital Admission (% of annual category visits)** | | | | | | | | | | | | | | | | |
| **2008** | 57,952 | 28.7 | 35,980 | 45.6 | 11,240 | 27.6 | 3,997 | 36.9 | 1,263 | 30.2 | 490 | 21.2 | 247 | 12.9 | 170 | 38.5 |
| **2009** | 62,557 | 27.9 | 36,570 | 44.3 | 12,249 | 27.1 | 4,229 | 33.0 | 1,417 | 31.5 | 411 | 17.3 | 310 | 12.5 | 172 | 12.0 |
| **2010** | 63,675 | 26.4 | 38,100 | 43.4 | 12,301 | 25.5 | 4,603 | 30.9 | 1,456 | 30.9 | 429 | 17.1 | 309 | 12.7 | 179 | 43.9 |
| **2011** | 68,151 | 26.4 | 39,127 | 43.0 | 13,280 | 25.5 | 5,127 | 30.8 | 1,461 | 29.8 | 343 | 13.0 | 376 | 12.6 | 173 | 37.5 |
| **2012** | 66,302 | 23.3 | 37,446 | 39.6 | 12,521 | 21.6 | 5,473 | 30.6 | 1,357 | 27.5 | 293 | 10.8 | 383 | 10.7 | 168 | 34.7 |
| **2013** | 57,303 | 19.8 | 32,752 | 34.4 | 10,986 | 19.7 | 5,724 | 28.8 | 1,422 | 24.4 | 243 | 8.2 | 359 | 9.4 | 171 | 35.0 |
| **2014** | 46,739 | 15.8 | 27,475 | 28.1 | 9,658 | 17.8 | 6,199 | 29.3 | 1,212 | 20.4 | 148 | 5.4 | 336 | 7.9 | 121 | 24.0 |
| **Overall** | 422,679 | 23.6 | 247,450 | 39.4 | 82,235 | 23.2 | 35,352 | 31.0 | 9,588 | 27.4 | 2,357 | 12.9 | 2,320 | 10.8 | 1,154 | 27.3 |

*MHD = Mental Health Diagnosis-Related*

*Note: The following diagnostic categories were excluded from this table: Psychiatric Examination and Miscellaneous/Other. They were excluded (1) because they represented a small proportion of MHD-related visits (0.7% and 1.3% on average, respectively) and (2) because they were non-specific categories and thus conveyed little information on known psychiatric disorders.*
